# Vitamin B12 deficiency in long-term metformin use and clinician awareness: a scoping review protocol

**DOI:** 10.1136/bmjopen-2025-101016

**Published:** 2025-07-22

**Authors:** Ian Parsonage, David Wainwright, Julian Barratt

**Affiliations:** 1 Health Sciences, University of Bath, Bath, England, UK; 2 Compass House Medical Centres, Brixham, UK; ^3^Aston University, Birmingham, England, UK

**Keywords:** Primary Health Care, Diabetes Mellitus, Type 2, Implementation Science

## Abstract

**Abstract:**

**Introduction:**

A relationship between long-term metformin use and vitamin B12 deficiency has been long discussed in the literature. Nonetheless, prior to 2022, there was no official guidance. In June 2022, the Medicines and Healthcare products Regulatory Agency (MHRA) published advice, stating that low vitamin B12 is now considered to be a common side effect. It advises checking levels in patients with symptoms of B12 deficiency, as well as monitoring those at risk of B12 deficiency.

Despite efforts to promote evidence-based practice, there is still a gap in the translation of research findings into policies and clinical practice. The above research has been shared widely in the academic and specialist diabetes literature over a prolonged period. The purpose of the scoping review is to explore what evidence is available regarding clinicians’ awareness of the association between metformin use and vitamin B12 deficiency in patients with type 2 diabetes mellitus, how this evidence is implemented into frontline clinical practice and what screening processes are recommended or exist.

**Methods and analysis:**

This is a protocol for a scoping review to be guided by the Joanna Briggs Institute (JBI) methodology for scoping reviews 20. The databases to be searched will include MEDLINE (accessed via PubMed), British Nursing Index, Google Scholar, Cochrane, Embase, Web of Science and Cumulative Index to Nursing and Allied Health Literature (CINAHL) (accessed via EBSCO), alongside searching for grey literature such as Electronic Theses Online Service (EThOS), DART European and Kings College London Research Portal. Titles and abstracts of articles will be reviewed by the authors. If articles are representative of the inclusion criteria, the articles will go through a full-text review by the authors. The results of the search and study inclusion/exclusion process will be reported and presented in a Preferred Reporting Items for Systematic Reviews and Meta-analyses flow diagram. Data will be extracted from papers, using the recommended JBI data extraction tool. The search will commence in August 2025, and the review is expected to be completed by November 2025.

The search will commence in August 2025, and the review is expected to be completed by November 2025.

**Ethics and dissemination:**

As this is a scoping review protocol that did not involve any human participants, human data or human tissue, no ethical approval was required. Our dissemination strategy includes peer review publication, presentation at conferences and with relevant stakeholders.

**STRENGTHS AND LIMITATIONS OF THIS STUDY**

- Comprehensive multidatabase and grey literature search strategy.
- Protocol follows Joanna Briggs Institute and Preferred Reporting Items for Systematic Reviews and Meta-Analyses Extension for Scoping Reviews guidance.
- Dual-reviewer screening and extraction will minimise selection bias.
- No critical appraisal is planned, consistent with scoping methodology.
- Restriction to English-language studies may omit relevant evidence.

## Introduction

 Diabetes is a serious and growing global health concern, according to the latest statistics. The global prevalence of impaired glucose tolerance was estimated at 7.5% (374 million) in 2019 and is projected to reach 8.0% (454 million) by 2030 and 8.6% (548 million) by 2045.^1^ The leading drug in the treatment of diabetes is metformin hydrochloride, with approximately 24.1 million items dispensed,^2^ with one study showing 83.6% of type 2 diabetes mellitus (T2DM) patients taking metformin.^3^

All guidelines, including the European Association for the Study of Diabetes and the American Diabetes Association (ADA), consider metformin a cornerstone and first-line treatment, along with lifestyle intervention, for managing hyperglycaemia in patients with T2DM.^4^ In the UK, metformin is the recommended first-line therapy for the treatment of T2DM in patients with normal renal function and is widely used.^5^

As far back as 1971, this effect was being studied with Tomkin^6^ reporting that approximately 30% of patients taking metformin do not properly absorb vitamin B12. A study published in 2010^7^ found that, compared with a placebo, the use of metformin 850 mg three times a day for 4 years was associated with a mean decrease in vitamin B12 concentration of almost 20%.

The first documented association was published in the late 1960s when annual serum vitamin B12 testing was already suggested as a valid screening measure for early detection of vitamin B12 deficiency in patients on long-term metformin therapy.^6 8^ However, definitive screening guidelines are lacking.

In 2021, the ADA Standards of Medical Care in Diabetes recommended considering a periodic assessment of vitamin B12 levels in patients with long-term metformin use, including those with pre-diabetes, peripheral neuropathy or anaemia.^9^ Infante undertook a ‘field of vision’ article into the link between vitamin B12 and metformin and suggested screening guidelines based on the current evidence and proposed a list of criteria for a cost-effective vitamin B12 deficiency screening in metformin-treated patients.^10^ Nonetheless, to date, no definite guidelines are available for the screening of vitamin B12 deficiency in patients taking metformin.

The known adverse drug reaction of vitamin B12 deficiency was recently reviewed for the brand leader Glucophage (metformin) within Europe with input from the MHRA.^11^ After this review, the MHRA has agreed that the product information for medicines containing metformin should be updated.

In June 2022, the MHRA published advice, which concluded that this side effect occurs more frequently than was previously thought.^11^ This has led to an update to the product information for all metformin-containing medications. The MHRA states that low vitamin B12 is now considered to be a common side effect, especially when taking high-dose or long-term metformin, affecting up to one in 10 people.^11^ It advises checking levels in patients with symptoms of B12 deficiency, as well as monitoring those at risk of B12 deficiency.^11^

Prior to an MHRA alert,^11^ there was no guidance issued on this matter in the UK.^12^ Currently, in the UK, the Clinical Knowledge Summary lists vitamin B_12_ deficiency as an adverse effect.^13^

The previous lack of formal screening guidelines for vitamin B12 deficiency in metformin-treated patients is likely due to longstanding uncertainty in the evidence base. One study^10^ highlighted key barriers, including inconsistent definitions of deficiency, limited agreement on reliable biomarkers and a lack of validated, cost-effective screening strategies. This study also noted that delayed symptom onset and unclear mechanisms of B12 malabsorption have contributed to clinical inertia. Despite clear associations between long-term metformin use and biochemical deficiency, the absence of high-quality prospective data has hindered policy development.^10^

Awareness of monitoring for adverse effects from medication has been shown to be poor in other similar cases. A recent study supported this view and highlighted how individual clinicians find it difficult to be aware of all the relevant, valid evidence, especially with many important practice changes linked to low-cost pharmaceuticals or non-pharmaceuticals.^14^

Despite efforts to promote evidence-based practice, there is still a gap in the translation of research findings into policies and clinical practice.^15^ The National Institute for Health and Care Research agrees and argues that when such interventions are implemented (or put into practice), the process is often challenging, unpredictable and typically slow.^16^

The research surrounding the risk of vitamin B12 deficiency with metformin use has been shared widely in the academic and specialist diabetes literature over a prolonged period. However, an MHRA alert had to be released to highlight this risk to clinicians surrounding this adverse effect. The purpose of the scoping review is to explore what evidence is available regarding clinicians’ awareness of the association between metformin use and vitamin B12 deficiency in patients with T2DM, how this evidence is implemented into front-line clinical practice and what screening processes are recommended or exist.

Scoping reviews are now seen as a valid approach in those circumstances where systematic reviews are unable to meet the necessary objectives or requirements of knowledge users.^17^ There now exists clear guidance regarding the definition of scoping reviews, how to conduct scoping reviews and the steps involved in the scoping review process.^18^ A scoping review may be applicable if authors do not always wish to ask such single or precise questions and may be more interested in the identification of certain characteristics/concepts in papers or studies and in the mapping, reporting or discussion of these characteristics/concepts. In these cases, a scoping review is the better choice.^19^

A scoping review was considered a more optimal choice over a systematic review for the question posed. The question covers several concepts and themes in which individual studies and reviews exist, but no mapping has taken place exploring these themes together.

A preliminary search of MEDLINE, the Cochrane Database of Systematic Reviews and Joanna Briggs Institute (JBI) Evidence Synthesis was conducted, and no current or underway systematic reviews or scoping reviews on the topic were identified.

### Review question

This scoping review is guided by a primary objective: to explore how awareness of vitamin B12 deficiency in patients on long-term metformin is understood, identified and translated into clinical practice by healthcare professionals. The review will address this through three connected subquestions, which will be analysed both individually and collectively to identify common themes and gaps in knowledge translation:

1. Clinician awareness—what is the extent of evidence regarding clinician awareness of vitamin B12 deficiency as a side effect of long-term metformin use in patients with type 2 diabetes?
2. Screening and monitoring practices—what are the existing screening and monitoring practices in primary care settings for early detection of vitamin B12 deficiency in patients on long-term metformin?
3. Implementation into clinical practice—how is the evidence on clinician awareness and screening practices implemented in frontline clinical practice for patients with type 2 diabetes on long-term metformin?

## Methods and analysis

### Methods

The proposed scoping review will be based on the JBI methodology for scoping reviews.^20^ Prior to this review, a thorough review of the literature will be undertaken. Systematic reviews may exist for each component of the title (vitamin B12 deficiency in patients on long-term metformin, clinician awareness of evidence-based practice, screening/early detection), but the scoping review will continue if no previous scoping reviews or studies have been undertaken looking at screening methods for metformin-related vitamin B12 deficiency or clinician awareness of vitamin B12 deficiency in patients on long-term metformin use. Therefore, this scoping review will be used to identify and analyse knowledge gaps.

**Figure 1:**
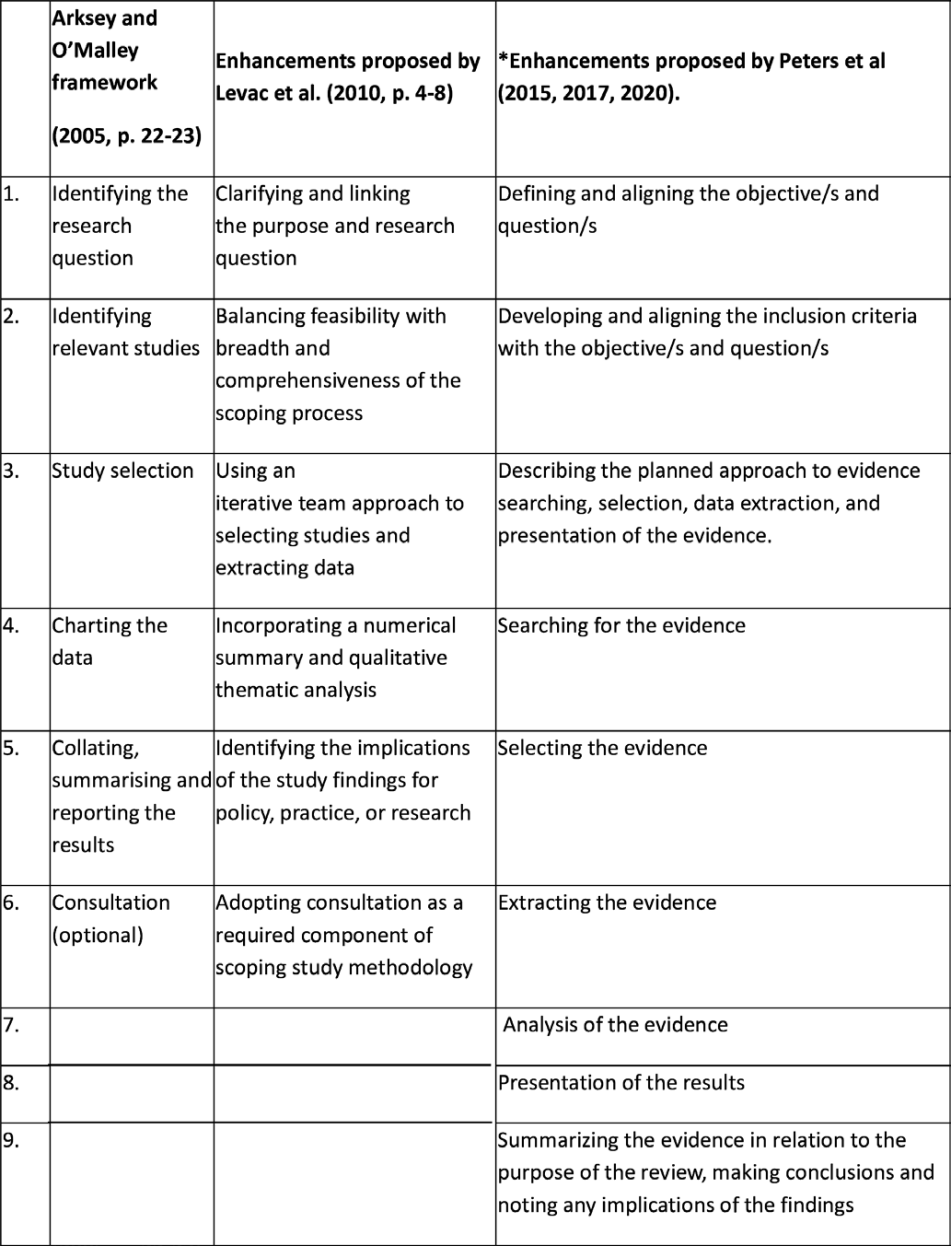
Scoping review methodology framework. This figure outlines the scoping review framework adapted from Arksey and O’Malley,^23^ with enhancements by Levac *et al* and Peters *et al.* It highlights the stages of review development from formulating the research question to summarising and reporting results.

The structure of this scoping review will be based on the framework proposed by Arksey and O’Malley and was revised by Peters^17^ (see figure 1). The methodological process will be based on the JBI approach. Preferred Reporting Items for Systematic reviews and Meta-Analyses Extension for Scoping Reviews (PRISMA-ScR) will be used for reporting of the scoping review as per the British Medical Jouranl (BMJ) guidelines.^21^

### Search strategy

The search strategy will aim to locate both published and unpublished studies. The text words contained in the titles and abstracts of relevant articles and the index terms used to describe the articles will be used to develop a full search strategy.

The search strategy, including all identified keywords and index terms, will be adapted for each database and/or information source. The reference list of all included sources of evidence will be screened for additional studies.

**Figure 2:**
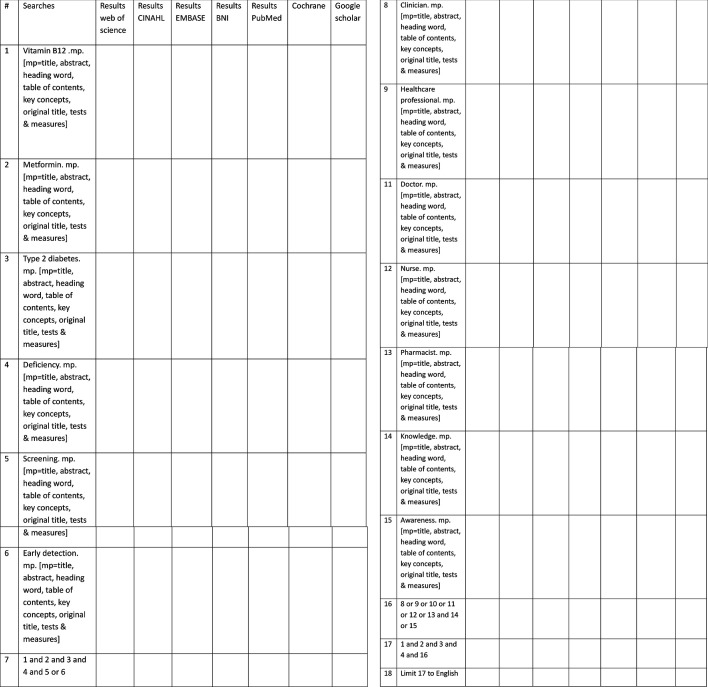
Search strategy structure. An overview of the search terms and Boolean operators used to develop the search strategy across selected databases, including MEDLINE, CINAHL, Embase and others. It reflects key terms related to vitamin B12 deficiency, metformin use and clinician awareness. BNI, British Nursing Index.

Only studies published only in English will be included. (See figure 2 for the search strategy). Resource constraints preclude translation and validated tools for non-English quality checking; therefore, only English-language papers will be included. Nevertheless, it is acknowledged that this may limit the scoping review’s rigour, as relevant evidence in non-English papers may be missed.

The databases to be searched include MEDLINE (PubMed), British Nursing Index (BNI), Google Scholar, Cochrane, Embase, Web of Science and CINAHL (EBSCO), alongside searching for grey literature such as EThOS, DART European and Kings College London Research Portal.

The databases to be included are selected to reflect the breadth of disciplines relevant to this topic, such as primary care, nursing, implementation science and pharmacology. MEDLINE and Embase provide coverage of biomedical and pharmacological literature, while CINAHL and BNI ensure nursing and allied health perspectives are captured. The inclusion of Web of Science and Google Scholar allows for wider citation tracking and grey literature, and Cochrane was added due to its relevance in synthesised healthcare evidence.

### Study/source of evidence selection

Following the search, all identified citations will be collated and uploaded into EndNote 21 Clarivate Analytics, Pennsylvania, USA. The full text of selected citations will be assessed in detail against the inclusion criteria. Only articles published since 1990 and written in English were eligible for inclusion in this review. Articles will be excluded if they did not pertain to clinician awareness of or screening of vitamin B12 deficiency in type 2 diabetes or metformin use. Titles and abstracts of articles will be reviewed by the authors. If articles are representative of the inclusion criteria, the articles will go through a full-text review by the authors. The results of the search and the study inclusion process will be reported in full in the final scoping review and presented in a PRISMA flow diagram.^22^

### Inclusion criteria

#### Study population

Participants over 18 with T2DM on long-term metformin. Registered healthcare professionals who prescribe or monitor patients with type 2 diabetes and on long-term metformin.

#### Concept

Papers need to look at vitamin B12 deficiency linked to long-term metformin use. Papers that explore clinician knowledge of side effects of metformin long-term. Papers where the full text is immediately available. Primary care professionals who are implementing screening protocols/monitoring protocol strategies for vitamin B12 deficiency in relation to long-term metformin use.

#### Context

Any healthcare settings (including international) in which registered healthcare professionals are responsible for the monitoring and prescribing of medication for patients with type 2 diabetes, including metformin. These articles must be published after 1990. Studies prior to 1990 will be excluded, as they may reflect a more paper-based healthcare systems with outdated diagnostic criteria and care models, making their findings less applicable to modern practice.

#### Keywords

Vitamin B12, Metformin, Deficiency, Knowledge, Healthcare professional, Clinician, Awareness, screening, primary care, and early detection.

### Types of sources

This scoping review will consider both qualitative and quantitative studies, including experimental and quasi-experimental study designs, including randomised controlled trials, non-randomised controlled trials, before-and-after studies and interrupted time-series studies. In addition, analytical observational studies, including prospective and retrospective cohort studies, case-control studies and analytical cross-sectional studies, will be considered for inclusion. This review will also consider descriptive observational study designs, including case series, individual case reports and descriptive cross-sectional studies for inclusion.

In addition, systematic reviews that meet the inclusion criteria will also be considered, depending on the research question.

### Data extraction

**Figure 3:**
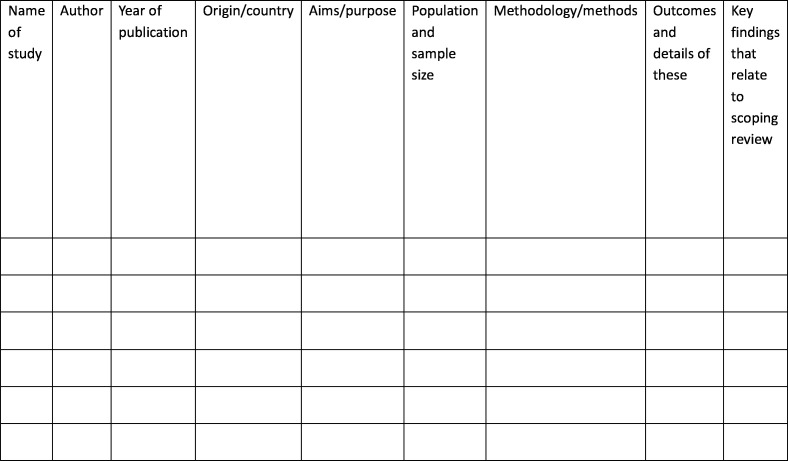
Draft data extraction instrument. A preliminary version of the charting tool used to extract data from included studies. It captures details such as study title, authorship, year, setting, study design, population, outcomes and key findings relevant to the review objectives.

Data will be extracted from papers included in the scoping review by the reviewer using a data extraction chart developed by the author (see figure 3). The data extraction was conducted by the lead author using a data extraction sheet that was developed after critical discussion. This sheet included information about the name of the study, author, year of publication, origin/country, aims/purpose, population and sample size, methodology/methods, outcomes and details of these key findings that relate to the scoping review. For extracting data from the discussion papers, the emphasis was placed on extracting apparent themes from the papers and then interpreting those themes using personal judgements.

The draft data extraction chart will be modified and revised as necessary during the process of extracting data from each included evidence source.

### Data analysis and presentation

The intention of the scoping review is to provide a map and summary of available evidence, not to synthesise results into a set of final estimates and findings to inform decision-making. The data gathered from the included studies will be discussed in a descriptive text.

### Study length

The search will commence in August 2025, and the review is expected to be completed by November 2025.

## Ethics and dissemination

As this is a scoping review protocol that did not involve any human participants, human data or human tissue, then no ethical approval was required. Our dissemination strategy includes peer review publication, presentation at conferences and relevant stakeholders.
