# Supplementary material for: Vitamin B12 deficiency in long-term metformin use and clinician awareness: a scoping review protocol
Source: BMJ Open. 2025 Jul 22;15(7):e101016. doi: 10.1136/bmjopen-2025-101016 (PMC12306209; doi:10.1136/bmjopen-2025-101016)
Supplement: Supplementary file 1 [file bmjopen-2025-101016.reviewer_comments.pdf]

PEER REVIEW HISTORY

BMJ Open publishes all reviews undertaken for accepted manuscripts. Reviewers are asked to complete a checklist review form and are provided with free text boxes to elaborate on their assessment. These free text comments are reproduced below.

ARTICLE DETAILS

Title (Provisional)

Vitamin B12 deficiency in long term metformin use and clinician awareness - a scoping review protocol

Authors

Parsonage, Ian; Wainwright, David; Barratt, Julian

VERSION 1 - REVIEW

Reviewer 1

Name CHENG, YIPING

Affiliation Shandong Provincial Hospital Affiliated to Shandong First Medical University, Department of Endocrinology

Date 04-May-2025

COI None

Here are several suggestions for this scoping review protocol on " Scoping review protocol-Scoping review Vitamin B12 deficiency in long term metformin use and clinician awareness":

1. Research Background and Scientific Rationale

- Suggestion: The authors mention the MHRA's 2022 alert on metformin-associated vitamin B12 deficiency but do not elaborate on why official guidelines were lacking before this (e.g., conflicting evidence, research gaps). It would strengthen the study's rationale to clarify this historical context.

- Reason: Clearly defining the knowledge gap and historical background would better justify the academic value of this review.

2. Research Questions and Objectives

- Suggestion: The three research questions (clinician awareness, screening practices, and implementation into clinical practice) should be more explicitly linked. Clarify whether each question will be addressed separately or through an integrated analysis.

- Reason: A tighter logical framework would enhance methodological rigor.

3. Methodological Refinements

- Database Selection: The authors list multiple databases (e.g., MEDLINE, CINAHL) but do not explain their selection criteria (e.g., coverage, disciplinary relevance). A brief justification should be added.

- Language Restriction: Limiting the review to English-language studies may introduce regional bias (e.g., excluding non-English research or guidelines). The potential impact of this limitation should be discussed.

- Timeframe Justification: The rationale for selecting 1990 as the cutoff year (e.g., widespread metformin use or advancements in B12 research) should be explained.

- Reason: Transparency in methodology is essential for reproducibility in scoping/systematic reviews.

Summary

This protocol is well-structured overall but could be strengthened by refining the scientific rationale, methodological details, and practical applications. With revisions, the study's rigor and utility would be significantly enhanced.

**Reviewer**	**2**
**Name**	**Didangelos, Triantafyllos**
**Affiliation**	**Aristotle University of Thessaloniki, Internal Medicine**
**Date**	**22-Jun-2025**
**COI**	**None**

This scoping review focuses on Vitamin B12 deficiency related to long-term metformin use and the awareness of clinicians regarding this issue. The role of Vitamin B12 deficiency in patients with Type 2 Diabetes Mellitus who have been on metformin for many years has not been thoroughly investigated. That is particularly significant because both conditions are known to cause neuropathy independently. Additionally, as the authors point out, a gap remains in translating research findings into clinical policies and practices. The methodology used in this scoping review is not only appropriate but also meticulously designed, which instills confidence in the robustness of the findings. Overall, this scoping review could be beneficial for everyday clinical practice and contribute to improved patient management and health outcomes.

VERSION 1 - AUTHOR RESPONSE

Reviewer: 1
Dr. YIPING CHENG, Shandong Provincial Hospital Affiliated to Shandong First Medical University, Shandong Clinical Research Center of Diabetes and Metabolic Diseases, Shandong

Key Laboratory of Endocrinology and Lipid Metabolism, Shandong Prevention and Control Engineering Laboratory of Endocrine and Metabolic Diseases, Shandong University
Comments to the Author:
Here are several suggestions for this scoping review protocol on " Scoping review protocol-Scoping review Vitamin B12 deficiency in long term metformin use and clinician awareness":

1. Research Background and Scientific Rationale
 - Suggestion: The authors mention the MHRA's 2022 alert on metformin-associated vitamin B12 deficiency but do not elaborate on why official guidelines were lacking before this (e.g., conflicting evidence, research gaps). It would strengthen the study's rationale to clarify this historical context.
 - Reason: Clearly defining the knowledge gap and historical background would better justify the academic value of this review.

An extra paragraph has been added to the 'background' to elaborate on why no official guidelines existed before 2022, which helps strengthens the rational as per the reviewers comment.

2. Research Questions and Objectives
 - Suggestion: The three research questions (clinician awareness, screening practices, and implementation into clinical practice) should be more explicitly linked. Clarify whether each question will be addressed separately or through an integrated analysis.
 - Reason: A tighter logical framework would enhance methodological rigor.

The research question was reformatted to allow for it be explicated linked and to make it clearer regarding the analysis with an explaining paragraph before the question.

3. Methodological Refinements
 - Database Selection: The authors list multiple databases (e.g., MEDLINE, CINAHL) but do not explain their selection criteria (e.g., coverage, disciplinary relevance). A brief justification should be added.
 - Language Restriction: Limiting the review to English-language studies may introduce regional bias (e.g., excluding non-English research or guidelines). The potential impact of this limitation should be discussed.
 - Timeframe Justification: The rationale for selecting 1990 as the cutoff year (e.g., widespread metformin use or advancements in B12 research) should be explained.
 - Reason: Transparency in methodology is essential for reproducibility in scoping/systematic reviews.

This has been justified in the methodology section as per the Editors comments as well.

Summary
This protocol is well-structured overall but could be strengthened by refining the scientific rationale, methodological details, and practical applications. With revisions, the study's rigor and utility would be significantly enhanced.

Reviewer: 2
Dr. Triantafyllos Didangelos, Aristotle University of Thessaloniki

Comments to the Author:
This scoping review focuses on Vitamin B12 deficiency related to long-term metformin use and the awareness of clinicians regarding this issue. The role of Vitamin B12 deficiency in patients with Type 2 Diabetes Mellitus who have been on metformin for many years has not been thoroughly investigated. That is particularly significant because both conditions are known to cause neuropathy independently. Additionally, as the authors point out, a gap remains in translating research findings into clinical policies and practices. The methodology used in this scoping review is not only appropriate but also meticulously designed, which instills confidence in the robustness of the findings. Overall, this scoping review could be beneficial for everyday clinical practice and contribute to improved patient management and health outcomes.

Reviewer: 1
If you have selected ‘Yes’ above, please provide details of any competing interests.: Not applicable

Reviewer: 2
If you have selected ‘Yes’ above, please provide details of any competing interests.: not applicable
